# Nephrin Signaling in the Podocyte: An Updated View of Signal Regulation at the Slit Diaphragm and Beyond

**DOI:** 10.3389/fendo.2018.00302

**Published:** 2018-06-05

**Authors:** Claire E. Martin, Nina Jones

**Affiliations:** Department of Molecular and Cellular Biology, University of Guelph, Guelph, ON, Canada

**Keywords:** podocyte, nephrin, phosphorylation, signal transduction, chronic kidney disease

## Abstract

Podocytes are a major component of the glomerular blood filtration barrier, and alterations to the morphology of their unique actin-based foot processes (FP) are a common feature of kidney disease. Adjacent FP are connected by a specialized intercellular junction known as the slit diaphragm (SD), which serves as the ultimate barrier to regulate passage of macromolecules from the blood. While the link between SD dysfunction and reduced filtration selectivity has been recognized for nearly 50 years, our understanding of the underlying molecular circuitry began only 20 years ago, sparked by the identification of *NPHS1*, encoding the transmembrane protein nephrin. Nephrin not only functions as the core component of the extracellular SD filtration network but also as a signaling scaffold *via* interactions at its short intracellular region. Phospho-regulation of several conserved tyrosine residues in this region influences signal transduction pathways which control podocyte cell adhesion, shape, and survival, and emerging studies highlight roles for nephrin phospho-dynamics in mechanotransduction and endocytosis. The following review aims to summarize the last 5 years of advancement in our knowledge of how signaling centered at nephrin directs SD barrier formation and function. We further provide insight on promising frontiers in podocyte biology, which have implications for SD signaling in the healthy and diseased kidney.

## An Overview of the Glomerular Filtration Barrier (GFB)

The kidneys are responsible for maintenance of blood volume, electrolyte balance, and blood pressure as well as filtering wastes from the blood, which are then excreted from the body as urine. When the kidneys fail, dangerous levels of fluid, electrolytes, and wastes accumulate, wreaking havoc on the body (1). Patients suffering from chronic kidney disease (CKD) display progressive renal dysfunction and irreversible kidney damage. Current CKD treatments focus on limiting disease progression as well as supportive therapies to treat consequences of suboptimal kidney function rather than the underlying cause of disease. Unfortunately, it is difficult to predict which patients will respond well to treatment, or those who will ultimately progress to kidney failure. With the rapidly increasing worldwide burden of CKD, it is of paramount importance to better understand the underlying mechanisms leading to kidney damage, to improve patient stratification and develop novel means to treat all stages of disease.

The nephron is the blood filtering subunit of the kidney and each is made up of a glomerulus, the site of primary filtration, as well as a network of tubules where this filtrate is concentrated and further refined before it passes to the bladder to await excretion (2). The GFB plays a critical role in not only filtering out solutes and excess water but also in retaining essential components, including cells, macromolecules, and proteins within the blood. Loss of GFB selectivity is a hallmark of kidney dysfunction and ultimately results in loss of these components into the urine (proteinuria).

**Figure 1:**
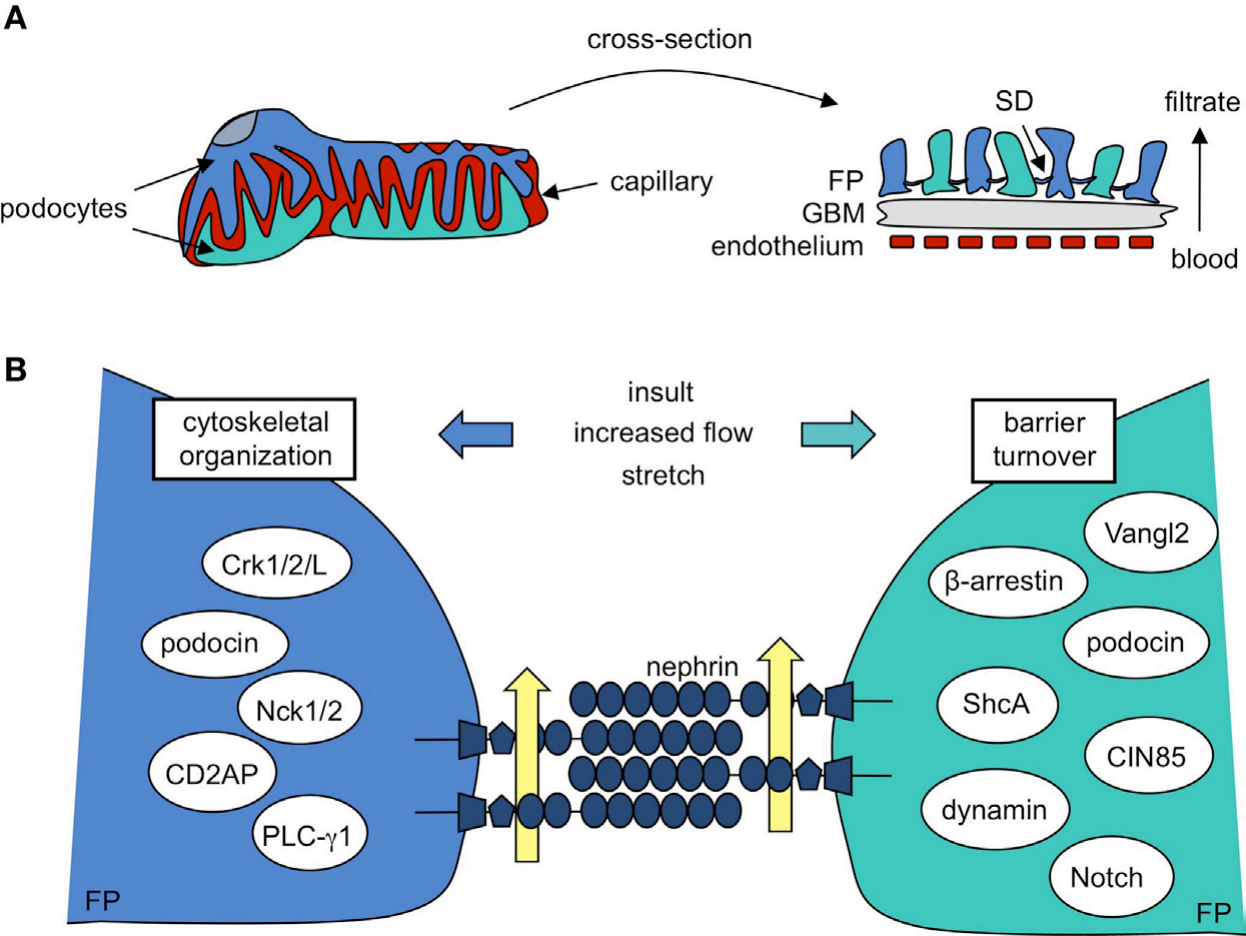
Podocyte protein nephrin is a central component in formation and maintenance of the slit diaphragm (SD). **(A)** Kidney podocytes wrap finger-like projections around the capillaries of the glomerulus, and these culminate in a network of interacting foot processes (FP). FPs contribute to the glomerular filtration barrier, which is also made up of fenestrated endothelium and the intermediating glomerular basement membrane (GBM). The SD, a specialized podocyte–podocyte junction found between interdigitating FPs, largely determines the size-selectivity of the barrier. **(B)** A closer look reveals the SD is a molecular sieve made up of nephrin molecules. The repetitive nature and precise patterning of extracellular nephrin–nephrin regions form pores that allow for discerning filtration of the blood (yellow arrows). Nephrin’s cytoplasmic region recruits a variety of signaling molecules that regulate cytoskeletal organization and FP shape as well as barrier turnover, each of which contributes to SD maintenance post-injury.

Glomerular dysfunction is a fundamental feature of kidney disease, and it is for this reason that the glomerulus remains the focus of sustained investigation into the pathogenesis and treatment of kidney disease (3). The glomerulus is a ball of tiny capillaries, each lined with a fenestrated endothelium and wrapped by highly specialized epithelial cells called podocytes (Figure 1A). The glomerular basement membrane (GBM), a compilation of proteins produced by podocytes and endothelial cells (4), lies at the interface of the two cell types. These three layers collectively constitute the GFB (5).

Central to filtration selectivity is the unique three-dimensional architecture of the podocyte. These highly arborized cells extend a series of processes from their cell body, which branch into an elaborate network of tertiary projections known as “foot processes” (6). Foot processes (FP) from adjacent podocytes interdigitate with each other to surround the glomerular capillaries and these ultimately act as the main barrier to loss of proteins and macromolecules into the urine. The extensive intercellular connections found between adjacent podocytes, which are referred to as filtration slits or slit diaphragms (SD), account for much of the size limitation of the GFB (Figure 1A) (7). The podocyte’s anionic glycocalyx, composed mainly of podocalyxin (8), provides additional charge-dependent resistance against the passage of negatively charged proteins. Together, their contribution toward both the size and charge basis for selective filtration positions podocytes as the key component of the GFB, and they are a primary target of injury in kidney disease (1, 2).

## The Unique Ultrastructure of the Podocyte

Recent advances in microscopy have allowed a detailed view of actin’s organization in the podocyte in both health and disease. Super-resolution imaging reveals a network of myosin IIA-containing contractile actin cables within podocyte cell bodies and major processes, which are also rich in intermediate filaments and microtubules. Conversely, myosin IIA-negative, non-contractile actin fibers populate podocyte FP (9), which are largely devoid of other cytoskeletal components.

The injured podocyte is characterized by retraction of FP into broader, more simplified structures in a process known as effacement, and loss of SD structures. Interestingly, super-resolution imaging has demonstrated that, after injury, the podocyte’s contractile acto-myosin network relocates from the major processes to the basolateral surface of the cell, manifesting as sarcomere-like structures juxtaposed to the GBM (9). Serial block face-scanning electron microscopy further demonstrates the formation of podocyte protrusions in the diseased glomerulus, which invade into disordered regions of the GBM (10). Importantly, these substructural changes are consistently observed in animal models with diverse genetic origins of disease, indicating that these alterations may represent common pathomechanisms. These findings suggest new details about the reorganization of the podocyte actin cytoskeleton during disease and the existence of yet-to-be investigated mechanisms that regulate this switch in podocyte architecture. One such mechanism may be the differential proteolytic cleavage of actin and SD proteins during disease, as was suggested by recent work (11).

## SD Assembly—Formation of a Molecular Sieve

The SD is a size-selective barrier that protects against loss of essential blood proteins, macromolecules, and cells into the urine during filtration. It is composed of molecules widely expressed in adherens (12) and tight junctions (13), such as P-cadherin, ZO-1, and Fat, as well as those predominantly expressed within podocytes alone, including nephrin (14, 15), nephrin-like (neph) 1 (16, 17) and podocin (18). The unique composition of the SD is central to its function and transition away from its distinct organization ultimately leads to barrier demise. Traditional SDs are often replaced by tight junctions during CKD progression (19, 20). This is potentially an attempt to bolster barrier function in the face of widespread effacement or to prevent the spread of immunogenic chemokines, which likewise lead to further damage. However, transition toward tight junctions creates a barrier that is virtually impermeable to passage of even fluid, prompting the reverse of the desired effect and ultimately leading to increased protein leakage and perpetuation of renal injury. This theory of tight junction-induced barrier dysfunction was recently confirmed in an experimental mouse model using podocyte-specific overexpression of the tight junction protein claudin-1, which *caused* SD destabilization and disorganization (21). These findings demonstrate not only the importance of the SD’s unique make-up for its function but also the importance of barrier flexibility in blood filtration.

The extracellular portion of the SD acts as a physical barrier and is comprised of two major components—nephrin and the related neph1. Each are members of the Immunoglobulin (Ig) protein superfamily. Nephrin’s large extracellular domain is made up of eight IgG-like motifs and a single fibronectin type 3 repeat (22). Neph1 similarly contains five IgG-like motifs in its extracellular portion (16). IgG domains can aggregate in *cis* and *trans* microclusters, and it is these IgG–IgG interfaces that are responsible for formation of the zipper-like SD meshwork that surrounds the glomerular capillaries, creating its sieve-like structure. Nephrin and neph1 also contain a single transmembrane domain and a short cytoplasmic tail. Contained within the intracellular region are several conserved residues that recruit cytosolic signaling partners that are likewise required for formation of the mature SD, which will be discussed in greater detail below.

The mature SD junction is not formed until the later stages of glomerular development (23). Podocytes originate in the S-shaped body as columnar epithelial cells containing, instead, apically localized tight junctions. During the early capillary loop stage, the apical membrane area of the podocyte expands and these junctions begin migrating baso-laterally. It is at this point that nephrin begins to be expressed (24). As the capillary loop stage proceeds, the apical domain of the podocyte continues to increase in size, and both nephrin and neph1 migrate toward the basal domain coincident with the start of podocyte process formation. Tight junctions containing nephrin form between these nascent processes (24). As the glomerulus continues to mature, podocytes extend additional processes as they wrap around the newly forming capillary loops and only then do the mature SD structures containing tyrosine phosphorylated nephrin and neph1 appear (25).

Nephrin/neph-like complex formation is an evolutionarily conserved adhesion module in which heterodimeric *trans* neph1-nephrin interactions occur between two distinct cell types, with one cell expressing nephrin and the other expressing neph1 (26, 27). In the glomerulus, however, interactions occur between adjacent podocyte cells which each express both nephrin and neph1 proteins, thereby allowing for nephrin–nephrin, neph–neph, and nephrin–neph pairings. The likely arrangement of nephrin and neph1 components in the SD was recently illuminated using high-resolution ultrastructural imaging (17). Unlike in other modules, nephrin and neph1 appear to minimally interact in podocytes. Instead, distinct segments of *trans* nephrin–nephrin and neph1–neph1 multimers largely comprise the SD in an approximate 2:5 ratio, with neph1 molecules spanning the lower part of the junction, closer to the GBM with a width of 23 nm, while nephrin molecules contribute to the apical region of the SD, with a width of 45 nm. These nephrin and neph1 complexes are spaced 7-nm apart, creating a two-tiered configuration, setting the SD apart from other nephrin–neph-like cell–cell adhesion modules.

Nephrin localization to the SD is dependent on its cytosolic interaction with podocin (28). Podocin (encoded by *NPHS2*) interacts with nephrin’s R1160 residue and directs its localization to lipid rafts at the cell surface (14, 15, 18, 29), enabling it to form the SD and perform its signaling functions. The podocin R138Q mutation is the most common *NPHS2* disease-causing variant (28, 30) and, similar to other *NPHS2* mutations, it disrupts podocin folding and glycosylation, resulting in severe congenital nephropathy associated with podocin’s retention in the endoplasmic reticulum (ER) (29, 31). Interestingly, expression of R138Q with nephrin in culture also results in nephrin’s retention in the ER (29), demonstrating a clear requirement for appropriate podocin trafficking in nephrin’s localization to the membrane.

Neph1 likewise depends on cytoplasmic interactions for its localization to the plasma membrane. Although neph1 has also been described to bind podocin (16), whether podocin mutations negatively influence neph1 localization remains to be investigated. Studies have alternatively focused on the role of the motor protein myosin 1c (Myo1c) in supporting neph1’s recruitment to and turnover at the plasma membrane (32, 33). Interestingly, Myo1c *inhibits* nephrin’s localization at the plasma membrane (32) in culture, a finding of unknown relevance *in vivo*.

Mouse knockout models have clearly demonstrated that loss of either nephrin (34, 35), podocin (36), or neph1 (37) expression is sufficient to disrupt SD formation and induce severe disease within days of birth and even *in utero*. Furthermore, over 250 distinct genetic mutations in *NPHS1* (38) and over 100 in *NPHS2* (39) have been identified to date. The overall importance of nephrin–podocin communication in development is further highlighted in instances of congenital nephrotic syndrome (CNS) in which single mutations in either *NPHS1* or *NPHS2* are benign in respective parents, but their digenic heterozygosity leads to congenital disease (40), a relatively uncommon phenomenon (41). Maintenance of nephrin and podocin expression throughout adulthood is also required (42, 43), as is their communication throughout life, which was recently demonstrated using a mouse model with inducible expression of a CNS-related *NPHS2* mutation (44). Adult animals induced to express the human-corresponding R138Q mutation, which causes retention of nephrin in the ER, rapidly develop nephrotic syndrome. This demonstrates an ongoing requirement for nephrin–podocin interaction throughout life and, more specifically, nephrin’s dependence on podocin for trafficking to the SD.

## Nephrin Phosphorylation Establishes a Signaling Platform

Beyond its role as a physical barrier, nephrin acts a central signaling platform within the podocyte, facilitated by an extensive network of cytoplasmic binding partners which have been identified over the past two decades (Figure 1B) (18, 29, 45–76). These proteins represent key components of diverse signaling cascades that affect podocyte polarity, cell survival, calcium mechano-signaling, membrane trafficking, and actin organization. Nephrin complexes with many of its binding partners *via* phosphorylation of various tyrosine and threonine residues found in specific binding motifs within its cytoplasmic region. The complex interplay between SD signaling and foot process morphology is accomplished in large part through cell signaling events centered at these conserved residues.

**Figure 2:**
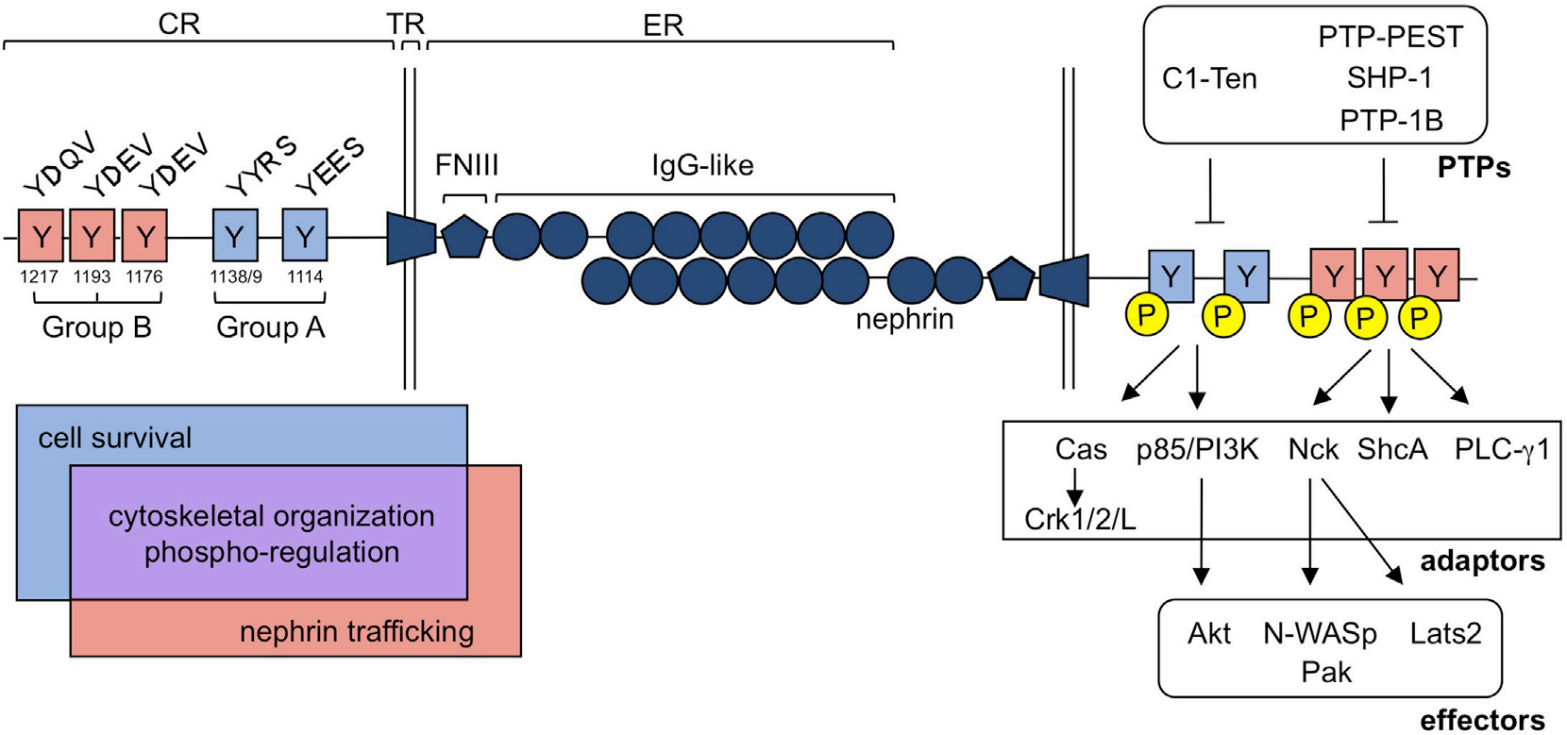
Nephrin tyrosine phosphorylation regulates a diverse group of signaling processes within the podocyte. Nephrin is a single pass transmembrane protein and contains a singular extracellular (ER), transmembrane (TR), and cytoplasmic (CR) region. Nephrin’s ER contains 1 Fibronectin-like III (FNIII) motif and eight Immunoglobulin (IgG)-like regions, which allow for homophilic interactions of nephrin molecules in *trans*. Tyrosine residues embedded in nephrin’s CR can be loosely classified into two categories, denoted as group A or B tyrosines, based largely on their flanking sequences. These consensus sequences are conserved and influence the adaptor molecules and downstream signaling effectors that can be recruited to nephrin upon their tyrosine phosphorylation. Interactions with each group contributes to cytoskeletal organization (through Cas-Crk/1/2/L, Nck-N-WASp/Pak and/or PLC-γ1) and nephrin’s phospho-regulation [through protein tyrosine phosphatases (PTPs)], denoted in purple. Alternatively, cell survival signaling appears to be largely restricted to signaling at group A residues (through p85/PI3K-AKT), denoted in blue, while nephrin trafficking is influenced by group B signaling (through ShcA), denoted in red.

The signaling pathways induced by nephrin tyrosine phosphorylation can be loosely defined based on their dependence on two different sets of residues. Group A tyrosines include Y1114 and Y1138/9, while group B tyrosines encompass Y1176, Y1193, and Y1217 (human nephrin numbering system) (Figure 2). These tyrosines often coordinate signal propagation independently of each other, but at times, can also work in concert. Both group A and B tyrosines can be phosphorylated by Src family kinases, including Src (77), Fyn (46, 47, 77), Lyn (77), and Yes (46, 77), although each residue may not be phosphorylated to the same extent (47, 48, 53, 62). Fyn kinase also directly binds group B phospho-tyrosine residues (46), which may provide better access to target tyrosine residues (78) as well as protection of these sites from de-phosphorylation (79).

Phosphorylation of group A tyrosines 1114 and 1138/9 induces binding of p85/PI3K (57, 80, 81), ultimately leading to activation of Akt and Rac1 and the recruitment of cofilin (57, 81). These residues also recruit Cas, which, once phosphorylated *via* group B-mediated signaling, allows for the engagement of Crk1/2/L (71, 73), which affects actin and focal adhesion dynamics within the podocyte.

The impact of phosphorylation of group B tyrosines 1176, 1193, and 1217 is the most widely characterized, potentially due to the commercial availability of phospho-specific antibodies for these sites (82). Phosphorylation at these sites results in the recruitment of Nck, PLC-γ1, and ShcA. Nck subsequently recruits both Pak (83, 84) and N-WASp (85), allowing for actin polymerization at nephrin (Figure 2).

Dysregulation of the actin cytoskeleton by mutations in proteins that link the SD to actin [α-actinin-4, FAT Atypical Cadherin (FAT)1, CD2AP, PLC-ε1, nephrin, and podocin] or that regulate actin polymerization [inverted formin 2, myosin 1e (Myo1e), Rho GTPase activating protein (ARHGAP) 24, Rho GDP Dissociation Inhibitor Alpha (ARHGDIA), and TRPC6] or podocyte contractility [myosin heavy chain 9, Myo1e, laminin subunit β2, integrin β4, integrin α3, Lamin A/C, tetraspanin (CD151) and several type IV collagens] give rise to spontaneous kidney disease in humans characterized by loss of filtration selectivity [reviewed recently Ref. (86)]. Connections between nephrin and actin, mediated both by or independently of group A and B tyrosines, allow for steady communication between the extracellular barrier and the core of the podocyte FP. Nephrin’s tyrosine phosphorylation appears to modulate two forms of actin in culture: lamellipodia formation, which is often associated with pathogenic FP effacement, and the growth of actin polymers (“tails” or “comets”) at nephrin, which are believed to stabilize foot process ultrastructure and the SD laterally. Lamellipodia formation appears to be modulated by p85/PI3K/Akt/Cas/Crk through phosphorylation of group A tyrosines (57, 71, 73, 81), while the production of actin tails at nephrin is dependent on phosphorylation of group B residues and recruitment of Nck (53, 85).

In addition to its several conserved tyrosine motifs, nephrin contains a S/TX_4_S/T region between residues 1120 and 1125, a consensus motif for serine/threonine phosphorylation. Protein kinase C alpha (PKCα), which also interacts with nephrin in a phosphorylation-independent manner, can phosphorylate nephrin at these residues (87, 88). This in turn enhances nephrin’s interaction with β-arrestin and ultimately induces its endocytosis in a clathrin (68, 89) and dynamin-dependent (90) fashion. Activation of this pathway has been characterized in diabetic (87), hypertensive (88), and acute (91) renal injury models, indicating that it may represent a central pathomechanism in disease. Exactly how phosphorylation of this residue is regulated is largely unknown, although there is some evidence that β-arrestin competes for nephrin with phospho-tyrosine dependent binding partners including Nck (88) as well as with podocin (52), a mechanism warranting further investigation.

## Phosphatase-Mediated Regulation of Nephrin Tyrosine Phosphorylation

Nephrin’s tyrosine phosphorylation is modulated by several protein tyrosine phosphatases that influence Fyn activation. De-phosphorylation of Fyn by the SHP-2 phosphatase (92), which also binds group B nephrin phospho-tyrosines (75), releases the intramolecular inhibition of Fyn, ultimately enhancing its kinase activity on nephrin (75). Conversely, the active site of Fyn (Y418) was found to be a substrate for the phosphatase PTP-PEST, and de-phosphorylation of this site leads to reduced Fyn activity, indirectly diminishing nephrin’s phosphorylation (67). Interestingly, Nck and ShcA, adaptor proteins known to bind group B phospho-tyrosines, work in a feed-forward mechanism by promoting activation of Fyn (76, 93). In the case of Nck, this is dependent on its ability to complex with nephrin and Fyn (93).

Tyrosine phosphatases can also act directly on nephrin (Figure 2). PTP-1B, which is upregulated in the puromycin aminonucleoside (PAN) model of membranous nephropathy (MN), can directly dephosphorylate rat tyrosine residues that correlate to the human sites 1193 and 1217 (67). Likewise, the SHP-1 phosphatase, which binds group A tyrosine residues, dephosphorylates tyrosines 1176, 1193, and 1217 (74) and its upregulation has been observed in instances of hyperglycemia and diabetes by several groups (74, 94–98). In podocytes, hyperglycemia induces a persistent increase in SHP-1 expression, due to epigenetic modification in the SHP-1 promoter (97), leading to insulin signaling resistance, podocyte dysfunction, and cell death. Interestingly, PTP-1B and SHP-1 both are unable to dephosphorylate nephrin’s Y1138 site (67, 74), indicating that these phosphatases likely do not exert their influence by modulating PI3K-Akt signaling, but rather through tyrosine residues that bind Nck, ShcA, and PLC-γ1.

C1-Ten is the most recently identified nephrin tyrosine phosphatase (99) and it uniquely targets the Y1114 and Y1138 motifs, disrupting nephrin-PI3K complex formation. Interestingly, C1-Ten is upregulated in diabetic nephropathy (DN) models and is associated with induction of podocyte hypertrophy (99). Increased levels of SHP-1 phosphatase have likewise been identified in models of DN (74, 94–98) resulting in reduced phosphorylation of Y1176, Y1193, and Y1217, and disruption of nephrin and Nck binding. These recent findings position global reductions in nephrin tyrosine phosphorylation as a mechanism of damage in DN, the most common form of CKD.

## Disruption of Nephrin Tyrosine Phosphorylation in Podocyte-Based Kidney Diseases

The role of nephrin signaling during disease has remained an intense area of interest since the discovery of reduced phosphorylation of group B tyrosine residues in instances of human disease and in various disease models. Alterations in phosphorylation of group B tyrosines has been described in minimal change disease (MCD) (100) and MN (101) as well as in the PAN (76, 82), protamine sulfate (54, 102), nephrotoxic serum (NTS) (75, 88, 103), and lipopolysaccharide (102) rodent injury models and type I diabetic Akita mice (74, 98). Similarly, altered levels of several phosphatases known to influence nephrin’s phospho-status have also been identified in instances of disease (104). Unfortunately, antibodies targeting group A tyrosines or nephrin’s 1120/1125 serine/threonine regions remain unavailable, making it difficult to fully understand the role of signaling at these sites.

Historically, there has been a lack of consensus on whether tyrosine phosphorylation of group A residues is up- or downregulated during disease, and whether these changes are associated with promoting podocyte damage or protection from injury. These discrepancies may be attributed to the use of multiple unique sets of phospho-specific antibodies by different groups, which variably recognize the three similar tyrosine-based motifs. However, the recent development of the nephrin-Y3F mouse model appears to have solidified an overall requirement for nephrin’s tyrosine phosphorylation in SD maintenance (102, 105). In this knockin model, all three group B tyrosine residues were converted to phenylalanine (denoted Y3F), a change which retains protein structure while preventing phosphorylation. Although nephrin-Y3F animals are born with no obvious impairments, they quickly develop progressive disease characterized by foot process effacement, GBM thickening and proteinuria. Furthermore nephrin-Y3F animals display a reduced ability to recover after damage induced by several acute injury models. This collective evidence supports the essential role of signaling through group B tyrosines in both SD barrier maintenance and podocyte repair.

Nck’s ability to recruit actin to nephrin may be central to the requirement of group B tyrosines in barrier stability. Inducible deletion of Nck in adult mice results in reduced nephrin tyrosine phosphorylation (93) and loss of actin recruitment to nephrin, leading to podocyte effacement and loss of SD structures (82), thereby supporting its role in barrier maintenance. Complex formation between nephrin and Nck is likewise interrupted in several acute injury models coincident with damage (88, 102) implicating a central importance for their interaction in SD function. Elegant biophysical studies have provided context for the overlapping nature of group B tyrosines on nephrin, all of which can bind Nck, and the iterative SH3 domains of Nck, all of which can bind N-WASp and activate Arp2/3 (106–108). Threshold levels of nephrin-Nck signaling appear to be necessary to effectively induce formation of biomolecular signaling nodes on synthetic membranes which promote actin polymerization (106, 107). Future studies in which these concepts are applied to cell systems may provide insight into the physiological relevance of these mechanisms and the unique requirement for multivalent interactions between nephrin, Nck, and actin in the podocyte.

## Endocytosis: An Emerging Focus in Podocyte Biology

As a function of their barrier role, SD components require regular replacement to maintain SD integrity. Endocytosis is the process by which cells internalize membrane-bound components including embedded surface receptors and their ligands. In the podocyte, two endocytic pathways have been identified: clathrin-mediated endocytosis (CME) and clathrin-independent endocytosis (CIE) (109). Generally, CME mediates the regular turnover of receptors (110) and appears to predominate within podocytes (111). Clathrin is recruited in the initiation stage of pit invagination, supported by various proteins including α-adaptin, synaptojanin and endophilin-1, and actin. Subsequently, the GTPase dynamin is recruited to the vesicle where it wraps and constricts the neck until the small membrane-encapsulated vesicle is cut free, completing its internalization. The majority of cargo endocytosed in this manner, about 95%, is believed to be recycled back to the plasma membrane and this pathway thereby defines a constitutive turnover mechanism in cells (112). The remaining 5% that is targeted for degradation may be damaged, requiring replacement by synthesizing mechanisms, or lost as a means to finesse receptor activity.

Reports characterizing the effects of podocyte-specific deletion of dynamin1/2 (111), synaptojanin (111), and endophilin (111) have clearly established the functional importance of endocytic machinery within podocytes. Evidence further indicates that dynamin can complex with nephrin indirectly during its own endocytosis (111, 113). Investigation of nephrin trafficking mechanisms has become a keen area of interest in recent years owing to the recognition of nephrin mislocalization in a broad range of human diseases including MN (101), CNS (114), steroid-resistant nephropathy (28), MCD (115), DN (63), and hypertensive nephropathy (88). However, relatively little is known about the specific mechanisms that dictate SD protein trafficking (112).

β-arrestin2 was the first nephrin binding partner identified to influence nephrin trafficking (52), over 10 years ago. It binds phosphorylated T1120/T1125 on nephrin’s cytoplasmic tail, which facilitates nephrin endocytosis in a CME fashion (88). β-arrestin mediated nephrin endocytosis is relatively well-characterized within podocytes and several pathways seem to converge on this mechanism. PKCα mediates nephrin phosphorylation of T1120/1125, a phenomenon that is enhanced by PLC-γ1 (87, 88), leading to β-arrestin recruitment and nephrin internalization. This pathway has been shown to be relevant in several diseases including in diabetes (116) and in response to the vascular protein angiotensin II (ANGII) (87, 88), which is upregulated with hypertension.

Activation of the planar cell polarity (PCP) pathway during glomerular development also appears to stimulate nephrin endocytosis *via* a clathrin/β-arrestin-dependent mechanism (68, 89). Disruption of Vangl2 activity, which is involved in the PCP pathway, results in increased surface expression of nephrin in podocytes, and this leads to disruption of glomerular maturation in mice (117). Notch activation likewise has been shown to induce nephrin internalization *via* a β-arrestin/dynamin-dependent, raft-independent route (90) and animals overexpressing activated Notch display proteinuria and damage that is associated with enhanced nephrin endocytosis and loss of SDs.

Phospho-regulation clearly allows for dynamic control of various signaling processes in the podocyte, downstream of nephrin, and emerging evidence indicates that this regulatory ability extends to modulation of nephrin endocytosis (109). However, there are conflicting reports regarding the potential role for site-specific nephrin phosphorylation events. Reduced phosphorylation of Y1193 has been shown to induce binding of β-arrestin and promote rapid removal of nephrin from the cell surface by CME (52), while phosphorylation of this same tyrosine promotes podocin binding to nephrin, which is proposed to localize nephrin to lipid raft microdomains where it is turned over at a slower rate by CIE (29, 109). Others have reported that mutation of Y1217 or compound mutation of Y1193/Y1176 decreases nephrin internalization (109, 113), and that enhanced dynamin-mediated phosphorylation of nephrin promotes its endocytosis (113). Interestingly, ShcA is the only nephrin phospho-tyrosine binding partner identified to date that actually binds tyrosine phosphorylated nephrin and contributes to nephrin internalization (76). ShcA, however, is expressed at low levels in the mature podocyte and its association with nephrin endocytosis appears to be a pathogenic one, as was recently uncovered in focal segmental glomerulosclerosis (FSGS), MCD, and immunoglobulin A nephropathies (76). It is likely then that other yet-to-be-identified phospho-nephrin binding partners are responsible for nephrin endocytosis in the healthy state during barrier maintenance.

## Disrupted Nephrin Trafficking—An Overlooked Mechanism of Hereditary Disease?

Despite the multitude of disease-causing *NPHS1* mutations identified, relatively few have been characterized for precisely how they affect nephrin function from a cell biology or cell signaling perspective. From the studies available, it appears that most of the *NPHS1* mutations lead to abnormal retention of nephrin in the ER, and thus failed trafficking to the cell surface (118, 119). This suggests that the majority of *NPHS1* mutations result in a loss-of-function of nephrin due to the inability for it to localize to the SD, which leads to the early-onset and severe disease often associated with *NPHS1* mutations (118). Assumedly, this also affects the ability of nephrin to be phosphorylated and there is some evidence that disruption of nephrin localization to the membrane results in its sub-maximal phosphorylation (29). Although less commonly reported, some mutations, such as V822M, do not appear to affect nephrin trafficking *to* the cell membrane, but rather they affect normal nephrin trafficking *from* the plasma membrane (120). This likewise causes CNS (121), indicating that bidirectional trafficking is important for normal nephrin function. Interestingly, although nephrin-V822M localizes normally to the cell membrane, it displays altered nephrin phosphorylation at group B residues and is unable to reorganize actin filaments (120). We question whether abnormal nephrin endocytosis may be an overlooked contributing factor in other instances of hereditary kidney disease.

## Emerging Evidence of SD-GBM Communication in Response to Mechanical Strain

In addition to the SD, foot process morphology and barrier integrity are also critically dependent on basally localized adhesive complexes, which secure the podocyte to the underlying basement membrane. Just as mutations in the lateral SD proteins nephrin and podocin are well-established to contribute to disease, mutations in α-actinin-4, integrin α3, type IV collagens, and laminins, all of which contribute to signaling within podocyte adhesomes, similarly result in congenital kidney disease (86).

The podocyte’s SD and focal adhesions are both stabilized through extensive connections with the underlying actin cytoskeleton. However, little is known about the mechanisms by which inter-compartment communication occurs. The Crk family of proteins, whose recruitment and activity are collectively modulated by both group A and group B tyrosine residues, influences lamellipodia formation and focal adhesion reorganization in podocytes in culture (71, 73). Arf6, a GTPase, was also recently shown to be activated downstream of nephrin tyrosine phosphorylation (122), leading to similar effects on lamellipodia and focal adhesion reorganization. A recent report showed that activation of β1 integrin can likewise induce nephrin tyrosine phosphorylation (123). These studies collectively link signaling between the basal cell compartment and the SD. Supporting the importance of compartmental crosstalk in barrier function, digenic predisposition to disease has been recently identified in individuals with heterozygous mutations in collagen IV and podocin (22, 124) or collagen IV and myosin1e. Further investigation characterizing SD-GBM communication is required to more fully elucidate the mechanisms by which FP maintain their unique structure and thereby the filtration barrier.

The podocyte is continually exposed to hemodynamic strain and shear stress, and dynamic signaling between the SD and focal adhesions is proposed to allow the cell to remain adhered to the GBM while maintaining its barrier function (125–127). It was recently revealed that nephrin signaling directly modulates Hippo mechano-signaling by regulating the stability of its pro-survival component YAP. Phosphorylated nephrin can recruit and sequester Lats2, its negative regulator, at group B tyrosines through an indirect mechanism involving Nck and WTIP (103), thereby limiting YAP’s degradation. During the initiation of NTS disease in mice, a reduction in nephrin tyrosine phosphorylation is observed at group B residues, reducing Lats2 inhibition, which ultimately leads to YAP’s degradation (103). Such activation of Hippo signaling has also been shown to induce podocyte cell death (128). The importance of YAP was further highlighted by the development of a podocyte-specific YAP knockout mouse, which developed kidney disease soon after birth, and reduced levels of YAP are associated with FSGS (129). Interestingly, expression of podocyte adhesive proteins localized to both its SD (nephrin, podocin, and α-actinin-4) and focal adhesions (integrins α3 αv, β1, β5), as well as components that are central players in Hippo mechanosensing (WWTR1, TEAD1, Yap1, and Lats2*)* were recently identified to be collectively regulated by the transcription factor WT-1. The presence of mutations in WT-1 in hereditary CNS has become more apparent in recent years (130–132) and this points at a potential central role for WT-1 in regulating the podocyte’s ability to respond to mechanical strain.

The significance of mechanical force sensation in the podocyte is further made evident by the discovery of human disease-causing mutations in *TRPC5* and *TRPC6* (50, 133), which encode the main calcium channels of the podocyte. Aberrant calcium signaling is linked to the pathogenic remodeling of the actin cytoskeleton that results in foot process effacement, although much debate remains about the ultimate impact of the activity of these channels on podocyte function (50, 134–139). Nephrin also appears to crosstalk with TRPC6, as previous studies have demonstrated that phosphorylation of nephrin Y1193 promotes its interaction with PLC-γ1, stimulating its activation and triggering calcium mobilization at TRPC6 channels (62, 140). Interestingly, nephrin may also impede calcium signaling by directly binding and inhibiting TRPC6 activity in a non-tyrosine-dependent fashion (140). In support of this, several disease-causing TRPC6 mutations leave the calcium channel unresponsive to nephrin inhibition in cell culture experiments (140), identifying an important role for nephrin-mediated regulation of TRPC6 in podocytes.

## Frontiers in Podocyte Biology—Toward Novel Treatments Targeting the Podocyte

Personalized medicine offers the promise of tailored therapeutics, reduced side effects and, ultimately, superior outcomes for patients. However, the movement toward more individualized treatment can only proceed once the molecular signatures underlying disease have been identified and characterized. With the diverse nature of renal pathologies, and the sparse treatment options available, the nephrology field is relying on basic research to uncover a more detailed understanding of renal disease etiologies in order to establish effective new therapies (141).

Given the importance of the SD in kidney function, the capacity for treatments to stabilize the SD barrier remains a keen area of interest for drug development. Expression of nephrin within the SD is essential for barrier maintenance throughout life (42, 44) and its aberrant endocytosis increasingly appears to be a central mechanism of disease (42, 44, 76, 88, 142). ANGII inhibition, which is commonly used in the treatment of hypertension, reportedly inhibits nephrin endocytosis, stabilizing the barrier and protecting mice from disease (88). Small molecule-facilitated preservation of the interaction between neph-1 and ZO-1, which is often lost at the SD during injury, has also been shown to promote barrier function in mice (143).

Since neph1 and nephrin are directly exposed to the circulating blood, and their roles predominantly have been characterized to be kidney-specific, they remain tempting targets for drug development (144). Nonetheless, both neph1 (145) and nephrin (34, 146–148) are expressed in a variety of other tissues and it is unclear whether pathologic non-renal effects of targeted treatments will arise. Recent studies have uncovered a role for nephrin in the trafficking and release of insulin from pancreatic beta cells (113, 149–151), and new evidence identifies previously unrecognized deficiencies in blood glucose regulation in CNS patients with known *NPHS1* mutations (150). This may have implications for the use of ANGII inhibition or other treatments that inhibit nephrin trafficking during renal disease, as it may cause negative consequences in the pancreas where a similar signaling pathway appears to be required for insulin release.

Targeting pathogenic actin polymerization (152, 153), abnormal TRPC5 and TRPC6 channel activation (136, 154, 155), aberrant integrin activation (156, 157), and depletion of laminin-521 in the GBM (158) remain other emerging prospective treatments, all of which have shown significant promise in animal models. GDC-0879, a B-RAF^V600E^ inhibitor, was also recently identified in a high-throughput screen of approved drugs to promote podocyte cell survival (159). Although the utility of this compound as a treatment for renal disease *in vivo* has not yet been explored, GDC-0879 has successfully been used in mouse models to inhibit tumor growth (160), making it an exciting candidate for future investigations in the kidney.

## Summary

Precise regulation of signaling at the SD is crucial for formation and maintenance of the GFB. Nephrin acts as the core of the SD and also as a signaling hub in the podocyte, modulating cell polarity, survival, adhesion, cytoskeletal organization, mechanosensing, and SD turnover. These roles have been revealed using a variety of cell and animal models that continue to improve in their sophistication as well as inferences from diverse human kidney diseases. The next frontier is to mobilize the insight gained from over two decades of “nephr(in)ology” research into novel therapies that target this unique indispensable protein and the molecules it communicates with.

## Author Contributions

CM and NJ contributed to the concept, design, and literature research for this review. CM prepared the figures and drafted the manuscript, in consultation with NJ.

## Conflict of Interest Statement

The authors declare that the research was conducted in the absence of any commercial or financial relationships that could be construed as a potential conflict of interest.
